# Susceptibility of Various Developmental Stages of the Fall Armyworm, *Spodoptera frugiperda*, to Entomopathogenic Nematodes

**DOI:** 10.3390/insects11120868

**Published:** 2020-12-07

**Authors:** Rajendra Acharya, Hwal-Su Hwang, Md Munir Mostafiz, Yeon-Su Yu, Kyeong-Yeoll Lee

**Affiliations:** 1 Department of Applied Biosciences, College of Agriculture and Life Sciences, Kyungpook National University, Daegu 41566, Korea; racharya2048@gmail.com (R.A.); bgtwo2@naver.com (H.-S.H.); munirmostafiz12@gmail.com (M.M.M.); 2 Institute of Plant Medicine, Kyungpook National University, Daegu 41566, Korea; 3 Quantum Bio Research Center, Kyungpook National University, Gunwi 39061, Korea; 4 Daedong Tech, Gyeongsan 38401, Korea; ysyu1973@naver.com; 5 Institute of Agricultural Science and Technology, Kyungpook National University, Daegu 41566, Korea

**Keywords:** biological control, alien invasive species, virulence assays, *Heterorhabditis indica*, *Steinernema carpocapsae*, *Steinernema longicaudum*, sustainable management

## Abstract

**Simple Summary:**

The fall armyworm, *Spodoptera frugiperda*, native to Central and South America, has recently invaded Africa and Asia, causing serious economic damage to various crops. The chemical control of fall armyworm is not effective due to the development of pesticide resistance as well as environmental contamination. Alternatively, biological control using natural enemies can be used successfully in a sustainable way. Entomopathogenic nematodes are soil-dwelling natural enemies for many underground pest insects including lepidopteran caterpillars. This study evaluates the virulence of several entomopathogenic nematodes on different developmental stages of fall armyworm. We found that all the larval and pupal stages of fall armyworm were highly susceptible to the entomopathogenic nematodes. Our study provides important information of entomopathogenic nematodes for the practical application of biological control of fall armyworm.

**Abstract:**

The fall armyworm, *Spodoptera frugiperda*, which is native to Central and South America, has recently invaded Africa and Asia, causing serious damage to various crops. Although management to date has been largely unsuccessful, entomopathogenic nematodes (EPNs) are a potential biological control agent that could be used to control the late larval and pupal stages of *S. frugiperda* that dwell under the ground. Here, we compared the virulence of seven EPNs against larval and pupal stages of *S. frugiperda.* In a Petri dish assay, both *Heterorhabditis indica* and *Steinernema carpocapsae* were highly virulent against younger larvae, whereas *S. arenarium* and *S. longicaudum* were highly virulent against older larvae. In contrast, *H. bacteriophora*, *Heterorhabditis* sp., and *S. kushidai* showed low virulence against all larval stages. In soil column and pot assays, *H. indica*, *S. carpocapsae*, and *S. longicaudum* were highly virulent against late larval and pupal stages compared with the other EPN species. Thus, *H. indica*, *S. carpocapsae*, and *S. longicaudum* are recommended for the biological control of *S. frugiperda*. Our study provides important information of EPNs for the practical application of biological control of fall armyworm.

## 1. Introduction

The fall armyworm, *Spodoptera frugiperda* (J.E. Smith) (Lepidoptera: Noctuidae), native to Central and South America, was introduced into Africa in 2016 and then rapidly spread into Asia, reaching Korea and Japan by 2019 [1,2,3,4]. The species is a polyphagous pest that causes significant economic loss in agriculture by damaging crops such as corn, rice, wheat, sorghum, beans, potato, and cotton [5,6,7]. The current *S. frugiperda* strains in Africa mostly infest corn crops, in which they reduce the yield by up to 57% [8].

Various pesticides have been applied in an attempt to control *S. frugiperda* in the environment. However, chemical control has not been efficacious because *S. frugiperda* has developed resistance to commonly used insecticides such as lambda-cyhalothrin, chlorpyrifos, spinosad, and lufenuron [9,10,11]. Although *Bacillus thuringiensis* (Bt) is often used as a biological control agent, *S. frugiperda* has also become resistant to Bt toxins [12,13]. Given the issues with resistance, and the fact that repeated pesticide use can negatively affect human health and environmental safety [14,15], it remains necessary to develop effective *S. frugiperda* control strategies, which could include biological control with natural enemies such as predators, parasitoids, and pathogens [8,16,17]. Indeed, biological control agents are highly recommended alternatives to hazardous chemical pesticides for the sustainable management of *S. frugiperda* [18].

Entomopathogenic nematodes (EPNs) can be used as biological control agents to control insect pests including various lepidopteran species [19,20,21]. In two EPN families, Heterorhabditidae and Steinernematidae, 116 species have been reported [22], some of which have been mass-reared and commercialized in various countries for the purposes of pest control [23,24,25,26,27,28]. The mode of action of EPNs, as previously established in many studies [29,30,31,32,33,34,35], is to penetrate the host insect through natural openings, such as the mouth, anus, and spiracles, and then release the mutualistic bacteria that they carry (*Photorhabdus* spp. and *Xenorhabdus* spp. in Heterorhabditidae and Steinernematidae, respectively); the bacteria reproduce and generate various metabolites and toxins that kill the host insect through septicemia or toxemia. However, the virulence of each EPN varies according to the species of host insect [36,37,38].

The virulence of EPNs not only depends on the host species but also their developmental stage [28,34,39,40,41,42]. For example, the virulence of *Steinernema carpocapsae* and *Heterorhabditis indica* was higher in the younger larval stage of *Spodoptera litura* [28] and the older larval stage of *Bradysia impatiens* [41]. Previous studies have suggested that EPNs have potential as biological control agents against *S. frugiperda* [19,21,43,44]; however, the virulence of EPNs at the different developmental stages of *S. frugiperda* has not yet been investigated.

In the present study, therefore, we evaluated the virulence of seven EPNs against larvae and pupae of *S. frugiperda*. Five of these EPNs, namely *H. bacteriophora*, *H. indica*, *S. arenarium*, *S. carpocapsae*, and *S. longicaudum,* are known to be highly virulent in many other insect species and are already used for pest control in the field [22,45,46,47,48]. In addition, the virulence of two newly collected and identified EPNs, namely *Heterorhabditis* sp. and *S. kushidai*, against *S. frugiperda* was tested for the first time. The virulence of *S. kushidai* to scarab beetle larvae has been reported, but its effects on lepidopteran larvae are not known [49,50]. The aim of our study is to provide the important information of EPNs for the practical application of biological control of fall armyworm.

## 2. Materials and Methods

### 2.1. Insects and Entomopathogenic Nematodes

*Spodoptera frugiperda* larvae were collected in August 2019 from a cornfield in Gyeongsan, Gyeongbuk province, Korea. The colony was maintained at 25 ± 1 °C and 60 ± 5% relative humidity (RH) under a 14:10 h light/dark cycle. Larvae were fed an artificial diet (Product number F9772; Frontier Scientific Services, Newark, DE, USA) prepared with 3.8 g of agar, 28.80 g of dry mix for lepidopteran insects, and 200 mL of distilled water (DW) [28].

Four EPN species, namely *H. bacteriophora*, *S. arenarium*, *S. carpocapsae*, and *S. longicaudum*, were obtained from Daedong Tech, Daegu, Korea. Both *H. indica* and *Heterorhabditis* sp. were isolated from Nepal, whereas *S. kushidai* was collected from Taejongdae in Busan, Korea. The EPNs were maintained using *Galleria mellonella* larvae as described by Woodring and Kaya [51]. Freshly harvested nematodes (not more than 2 weeks old) were used in the experiments.

### 2.2. Nematode Infection of S. frugiperda Larvae

#### 2.2.1. Effect of Larval Developmental Stage and Exposure Time on EPN Virulence

First- to sixth-instar larvae were used in the experiments. Each stage of larvae was separately placed in a Petri dish (*n* = 5 per 90 mm Petri dish) lined with 90 mm diameter qualitative filter paper (Hyundai Micro, Seoul, Korea) and containing 2.5 g of *Spodoptera* artificial diet. The virulence of all seven EPN species was determined by adding 250 infective juveniles (IJs) in 1 mL of DW to the surface of the filter paper in each dish. In the control treatment, 1 mL of DW only was applied. The Petri dishes were then sealed with Parafilm (Bemis Company Inc., Neenah, WI, USA) and kept at 25 ± 1 °C and 60 ± 5% RH. Larval mortality was determined at 24 h intervals for up to 72 h post-treatment. Each treatment contained ten larvae and the experiment was repeated three times on different dates.

#### 2.2.2. Efficacy of EPNs against *S. frugiperda* Larvae in a Soil Column Assay

A soil column assay was performed as described by Acharya et al. [28]. Glass cylinders (12 cm in height × 3 cm in diameter) were filled to the 10 cm mark with autoclaved nursery media (Punong, Gyeongju, Korea) containing 68% coco peat, 7% perlite, 14.729% peat moss, 3% vermiculite, 7% zeolite, 0.243% fertilizer, 0.024% pH regulator, and 0.004% moisture. A sixth-instar *S. frugiperda* larva was then added to the cylinder and covered with 2 cm of soil. Subsequently, 5 mL of DW containing 600 IJs was applied to the soil medium. The control glass cylinder received only DW. Both ends of the cylinders were covered with mesh netting before being placed vertically in an environmental chamber at 25 ± 1 °C and 60 ± 5% RH. Mortality was recorded at 24 h intervals for up to 72 h post-treatment. Each treatment contained three larvae and the experiment was repeated three times on different dates.

#### 2.2.3. Efficacy of Selected EPNs against *S. frugiperda* Larvae in a Pot Assay

Corn seedlings were grown in plastic pots (9 cm in diameter and 9 cm deep) with nursery media (Punong, Gyeongju, Korea). *Spodoptera frugiperda* sixth-instar larvae (*n* = 15) were placed into three corn pots (30 days old after transplantation) within an acre cage (40 × 40 × 50 cm). Subsequently, *H. bacteriophora*, *H. indica*, *S. arenarium*, *S. carpocapsae*, and *S. longicaudum* were applied to each pot separately at a density of 25 IJs/cm^2^. DW alone was applied to the control pot. Larval mortality was recorded for 5 days post-treatment. The experiment was repeated three times on different dates.

### 2.3. Nematode Infection of S. frugiperda Pupae

Five-day-old *S. frugiperda* pupae (*n* = 5) were placed in a 90 mm Petri dishes filled with 10 g of nursery media (Punong, Gyeongju, Korea). The effects of all seven EPN species were determined by adding 600 IJs in 5 mL of DW to each dish. DW alone was added to the control dishes. The Petri dishes were then sealed with Parafilm (Bemis Company Inc.) and kept at 25 ± 1 °C and 60 ± 5% RH. The adult eclosion rate was recorded for 5 days post-treatment. Each treatment contained ten pupae and the experiment was repeated three times on different dates.

### 2.4. Assessment of EPN Reproduction Rate in Different Larval Stages of S. frugiperda

The EPN reproduction rate was assessed using the White trap method [52]. Five EPN species, namely *H. bacteriophora*, *H. indica*, *S. arenarium*, *S. carpocapsae*, and *S. longicaudum*, were separately added (250 IJs/larva) to 90 mm Petri dishes lined with filter paper containing first- to sixth-instar larvae separately (*n* = 5) and 2.5 g of *Spodoptera* artificial diet. For each EPN species, fifteen cadavers were individually placed on the White trap after 4 days of nematode infection and harvested nematodes were counted for 15 days using a stereo microscope (SZ-ST; Olympus, Tokyo, Japan).

### 2.5. Statistical Analysis

Statistical Analysis System version 9.4 (SAS Institute, Inc., Cary, NC, USA) was used to perform all analyses [53]. The effects of EPN species, nematode exposure time, and larval developmental stage on the mortality of *S. frugiperda* larvae were determined using three-way ANOVA. Similarly, two-way ANOVA was used to determine the effects of EPN species and larval developmental stage on nematode reproduction in *S. frugiperda* larvae. Abbott’s formula [54] was used to calculate the corrected mortality. Lethal median time (LT_50_) was calculated in SAS using the corrected mortality. Tukey’s test, with significance set at *p* < 0.05 [55], was used to determine the differences among treatments. All data are represented graphically as means ± standard errors for each treatment.

## 3. Results

### 3.1. Effect of EPNs on the Mortality of Different S. frugiperda Larval Stages

**Figure 1:**
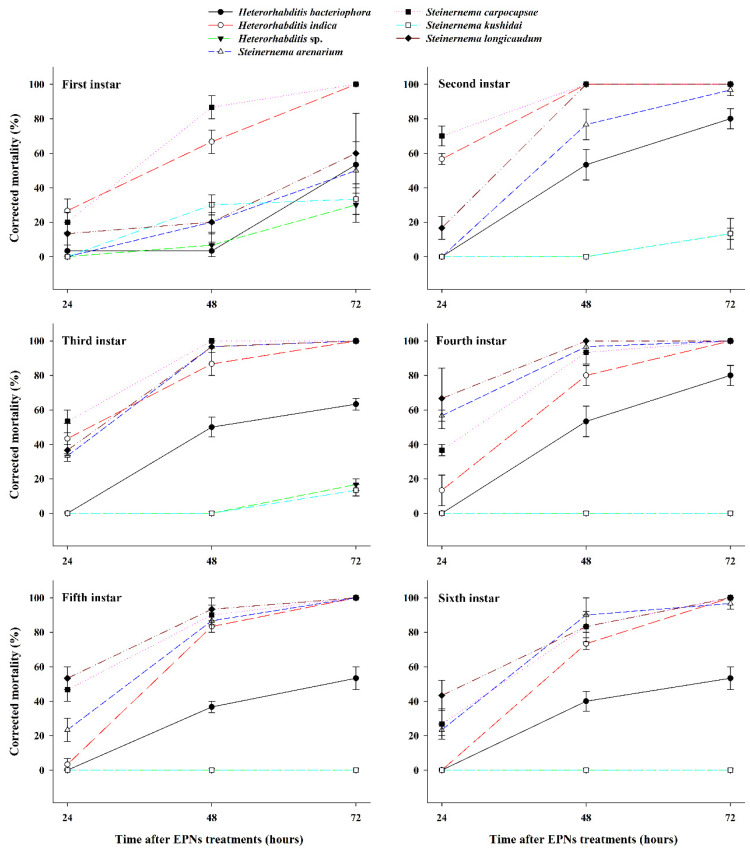
Corrected mortality caused by seven entomopathogenic nematodes (EPNs) in the larval stages of *Spodoptera frugiperda*. EPNs at a concentration of 50 infective juveniles per larva were applied to different larval stages of *S. frugiperda* and mortality rates were determined after 24, 48, and 72 h.

*Spodoptera frugiperda* larval mortality at different development stages was compared among EPN treatments and treatment durations (Figure 1). Larval mortality differed significantly among the six larval stages, and it was significantly affected by EPN species and exposure time (24, 48, and 72 h) (Table 1). In first-instar larvae, *S. carpocapsae* and *H. indica* caused 100% mortality at 72 h post-treatment; *S. longicaudum*, *H. bacteriophora*, *S. arenarium*, *S. kushidai*, and *Heterorhabditis* sp. caused 60%, 53%, 50%, 33%, and 30% mortality, respectively. In second-instar larvae, 100% mortality was caused by *H. indica*, *S. carpocapsae*, and *S. longicaudum*, whereas *S. arenarium* and *H. bacteriophora* caused 77% and 53% mortality, respectively, at 48 h post-treatment; *Heterorhabditis* sp. and *S. kushidai* did not cause any mortality. In third-instar larvae, 100% mortality was obtained with *H. indica*, *S. arenarium*, *S. carpocapsae*, and *S. longicaudum* at 72 h post-treatment; *H. bacteriophora* caused 63% mortality, *Heterorhabditis* sp. caused 17% mortality, and *S. kushidai* caused 13% mortality under the same conditions. In fourth- and fifth-instar larvae, similar mortality patterns were observed, i.e., *H. indica*, *S. arenarium*, *S. carpocapsae*, and *S. longicaudum* produced 100% mortality at 72 h post-treatment. In sixth-instar larvae, 100% mortality was achieved with *H. indica* and *S. carpocapsae* at 72 h post-treatment, whereas *S. arenarium*, *S. longicaudum*, and *H. bacteriophora* caused 97%, 93%, and 53% mortality, respectively. In the fourth, fifth, and sixth larval stages, *Heterorhabditis* sp. and *S. kushidai* did not cause mortality at any duration.

LT_50_ values for *H. bacteriophora*, *H. indica*, *S. arenarium*, *S. carpocapsae*, and *S. longicaudum* treatments against different *S. frugiperda* larval stages were calculated (Table 2). Since the mortality caused by *Heterorhabditis* sp. and *S. kushidai* was <50% up to 72 h post-treatment, LT_50_ values for these EPNs were not calculated. Among the remaining EPNs, the LT_50_ values of *S. carpocapsae* were lower in the first-instar (32 h; χ^2^ = 37.85, df = 1, *p* = 0.0001), second-instar (23 h; χ^2^ = 0.00, df = 1, *p* = 0.9998), and third-instar (24 h; χ^2^ = 0.00, df = 1, *p* = 0.9998) larvae. In contrast, the LT_50_ values of *S. longicaudum* were lower in the fourth-instar (23 h; χ^2^ = 0.00, df = 1, *p* = 0.9998), fifth-instar (23 h; χ^2^ = 19.70, df = 1, *p* = 0.0001), and sixth-instar (27 h; χ^2^ = 0.00, df = 1, *p* = 0.9998) larvae.

### 3.2. Efficacy of EPNs against S. frugiperda Larvae in the Soil Column Assay

**Figure 2:**
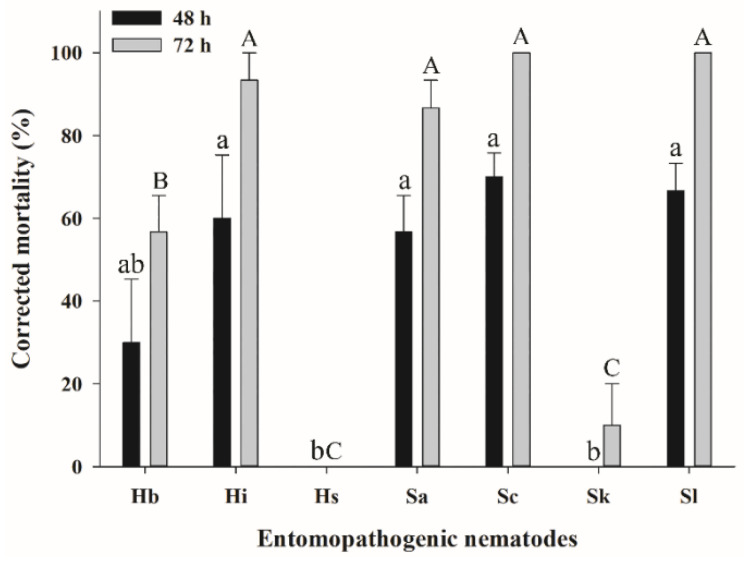
Corrected mortality of *Spodoptera frugiperda* in a soil column assay. A sixth-instar larva (*n* = 1 per glass cylinder) of *S. frugiperda* was treated with entomopathogenic nematodes (EPNs) (*n* = 600 infective juveniles) and mortality was determined 48 and 72 h after treatment. EPNs: *Heterorhabditis bacteriophora* (Hb), *H. indica* (Hi), *Heterorhabditis* sp. (Hs), *Steinernema arenarium* (Sa), *Steinernema carpocapsae* (Sc), *Steinernema kushidai* (Sk), and *Steinernema longicaudum* (Sl). The same small and capital letters above a bar indicate that there was no significant difference in mortality between EPNs at 48 and 72 h, respectively (*p* > 0.05, Tukey’s test).

In the soil column assay, the mortality rates of sixth-instar *S. frugiperda* larvae were determined 48 and 72 h after treatment with all seven EPN species at 600 IJs/Petri dish (Figure 2). The interaction between EPN species and post-treatment time for nematode infection was significant (F = 12.73; df = 12, 62; *p* < 0.0001). Mortality was observed after 48 h, and the rate was directly proportional to the nematode exposure time. Higher mortality occurred at 72 h post-treatment, with 100% mortality caused by *S. carpocapsae* and *S. longicaudum*; in contrast, *H. indica*, *S. arenarium*, *H. bacteriophora*, and *S. kushidai* achieved 93%, 87%, 57%, and 10% mortality, respectively, whereas *Heterorhabditis* sp. did not cause mortality over the experimental period.

### 3.3. Efficacy of EPNs against S. frugiperda Larvae in the Pot Assay

**Figure 3:**
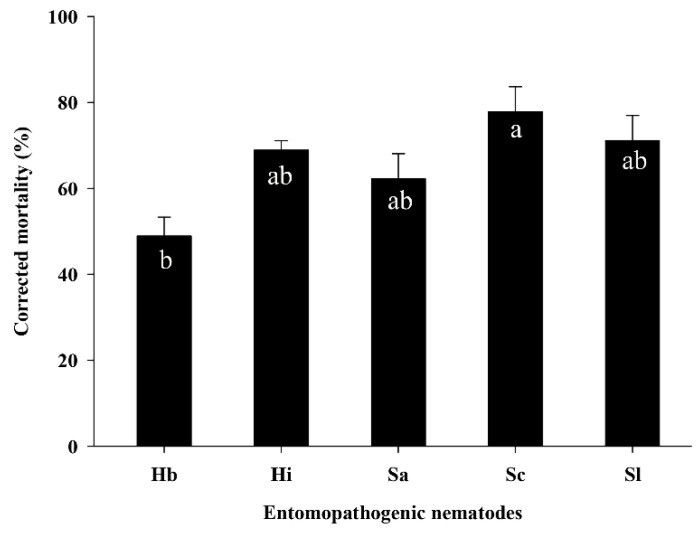
Corrected mortality of *Spodoptera frugiperda* in the corn pot assay. Sixth-instar larvae (*n* = 5 per pot) of *S. frugiperda* were treated with entomopathogenic nematodes (EPNs) (*n* = 25 infective juveniles/cm^2^) in a corn pot and mortality was determined 5 days after treatment. EPNs: *Heterorhabditis bacteriophora* (Hb), *Heterorhabditis indica* (Hi), *Steinernema arenarium* (Sa), *S. carpocapsae* (Sc), and *S. longicaudum* (Sl). The same letters in a bar indicate that there was no significant difference in mortality between EPNs (*p* > 0.05, Tukey’s test).

The efficacy of five EPN species, i.e., *H. bacteriophora*, *H. indica*, *S. arenarium*, *S. carpocapsae*, and *S. longicaudum*, against the sixth-instar larvae of *S. frugiperda* was evaluated at 25 IJs/cm^2^ for 5 days in corn-growing pots (Figure 3). The mortality rates of *S. frugiperda* larvae differed significantly according to EPN species (F = 4.68; df = 4, 14; *p* < 0.0219). Among the EPNs, *S. carpocapsae* caused higher mortality (78%) compared with the mortality caused by *S. longicaudum* (71%), *H. indica* (69%), and *S. arenarium* (62%). Meanwhile, *H. bacteriophora* caused significantly lower mortality (49%) among the tested nematodes.

### 3.4. Effect of EPNs on the Adult Eclosion Rate of S. frugiperda Pupae

**Figure 4:**
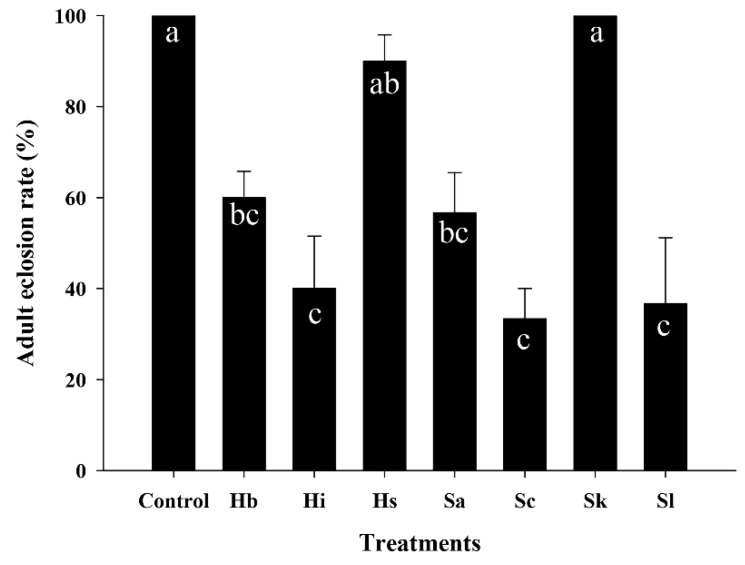
Effect of entomopathogenic nematodes (EPNs) on the adult eclosion rate of *Spodoptera frugiperda* pupae. Pupae (*n* = 5 per Petri dish) of *S. frugiperda* were treated with EPNs (600 infective juveniles) for 5 days. EPNs: *Heterorhabditis bacteriophora* (Hb), *H. indica* (Hi), *Heterorhabditis* sp. (Hs), *Steinernema arenarium* (Sa), *S. carpocapsae* (Sc), *S. kushidai* (Sk), and *S. longicaudum* (Sl). The same letters in a bar indicate that there was no significant difference in the adult eclosion rate between EPNs (*p* > 0.05, Tukey’s test).

The effects of all seven EPN species on the adult eclosion rate of *S. frugiperda* pupae were evaluated (Figure 4). The adult eclosion rates of *S. frugiperda* pupae differed significantly among EPN species (F = 9.11; df = 6, 20; *p* < 0.0004). Relative to the control, significantly lower adult eclosion rates were observed with *S. carpocapsae* (33%), *S. longicaudum* (37%), *H. indica* (40%), *S. arenarium* (57%), and *H. bacteriophora* (60%) treatments. Eclosion rates were higher with *Heterorhabditis* sp. (90%) but were not affected by *S. kushidai*.

### 3.5. EPN Reproduction Rate in Different Larval Stages of S. frugiperda

The reproduction rates of five EPNs, i.e., *H. bacteriophora*, *H. indica*, *S. arenarium*, *S. carpocapsae*, and *S. longicaudum*, were compared among the various instars of *S. frugiperda*, with EPNs at a density of 50 IJs/larva (Table 3). The interaction between EPN species and *S. frugiperda* larval stage was significant (F = 8.96; df = 20, 89; *p* < 0.0001). The EPN reproduction rate was proportional to the *S. frugiperda* developmental stage, with higher reproduction rates observed for *H. indica*, *S. carpocapsae*, and *S. longicaudum* in sixth-instar larvae.

## 4. Discussion

The virulence of EPNs depends on their innate characteristics as well as the conditions of their hosts [42,47,48,56]. Our results revealed that EPN virulence differed significantly among EPN species, exposure durations, and the developmental stages of *S. frugiperda*. Among the seven tested EPNs, *H. bacteriophora*, *H. indica*, *S. arenarium*, *S. carpocapsae*, and *S. longicaudum* were virulent to varying extents in all *S. frugiperda* larval stages, whereas *Heterorhabditis* sp. and *S. kushidai* caused mortality only in early instars. EPN virulence was dependent on the developmental stage of the host *S. frugiperda*: both *H. indica* and *S. carpocapsae* were highly virulent in younger instars (e.g., first-, second-, and third-instar larvae), whereas both *S. arenarium* and *S. longicaudum* were highly virulent in older instars (e.g., fourth-, fifth-, and sixth-instar larvae). The body lengths of *H. indica* and *S. carpocapsae* were 500 and 581 µm, respectively, whereas those of *S. arenarium* and *S. longicaudum* were 1082 and 832 µm, respectively; thus, the body size of an EPN species seems to be associated with virulence at different developmental stages of the host insect. Our results are consistent with previous studies. For example, Park et al. [34] reported that larger nematodes were able to penetrate larger host insects. In addition, Yan et al. [42] reported that *S. arenarium* was more virulent against third- and fourth-instar larvae of *S. litura* than against second-instar larvae. However, Patil et al. [56] reported that the virulence of *S. carpocapsae* did not differ between the second and fourth instars of *Mythimna separata* larvae.

The reproduction rate of EPNs is also influenced by the different developmental stages of host insects [34]. Our results indicate that the reproduction rates of five EPNs were significantly dependent on the larval stage of *S. frugiperda*. Specifically, the reproduction rates of *H. indica*, *S. carpocapsae*, and *S. longicaudum* were higher in sixth-instar larvae than in younger developmental stages. This result is consistent with a previous study that showed the reproduction rates of *H. indica* and *S. longicaudum* were higher in the fifth and sixth instars of *S. litura* [28].

Many studies report that lepidopteran pupae are also susceptible to EPNs [39,42,56,57,58,59]. For example, Kaya and Hara [57] reported that lepidopteran pupae are susceptible to *Steinernema feltiae*, which caused at least 47% mortality with 200 IJs. Similarly, Fuxa et al. [39] reported that *S. feltiae* successfully killed 7–20% of *S. frugiperda* pupae. The present study showed that three EPN species, namely *H. indica*, *S. carpocapsae*, and *S. longicaudum*, were highly lethal to *S. frugiperda* pupae, whereas *S. kushidai* was not effective. Yan et al. [42] also reported that *S. carpocapsae* and *H. indica* were highly virulent against *S. litura* pupae.

Previous studies have reported that *S. carpocapsae* is highly effective as a biological control against lepidopteran pests [28,36,60]. The present study also indicated that *S. frugiperda* larvae were most susceptible to *S. carpocapsae*, although other EPNs, such as *H. indica*, *S. arenarium*, and *S. longicaudum*, were also effective in the soil column and pot assays. Campbell et al. [61] reported that *S. carpocapsae* remains in the upper layer of soil to a large extent, whereas *H. bacteriophora* is uniformly distributed to a depth of up to 8 cm in the soil. Moyle and Kaya [62] and Pathak et al. [63] reported in their respective studies that more than 90% of applied *S. carpocapsae* and *S. frugiperda* larvae remain in the soil (2.5–5 cm depth). To validate the present results, the control efficacy of *S. carpocapsae*, as well as other EPNs, against *S. frugiperda* should be evaluated in a field study. Soil factors such as texture, moisture, pH, and depth also affect the control efficacy of EPNs. In general, higher efficacy of EPNs was observed in sandy soil than clay soil [64]; however, other studies reported that EPN efficacy was higher in clay soil than sandy soil [65,66]. Further investigation is required to understand the effects of soil parameters on EPN efficacy.

## 5. Conclusions

In conclusion, *S. frugiperda* was highly susceptible at larval and pupal stages to various EPNs including *S. carpocapsae*, *H. indica*, and *S. longicaudum*. These EPNs, therefore, could potentially be used as biological control agents to sustainably manage the overlapping generations of *S. frugiperda* in the environment.

## Figures and Tables

**Table 1:** ANOVA parameters for the individual effects of larval stage, post-treatment time, entomopathogenic nematode species, and their associated interactions on the mortality of *Spodoptera frugiperda* larvae over 72 h.

Source	DF	F Value	*p*
Larval stage (L)	5	20.29	0.0001
Post-treatment time (T)	2	579.6	0.0001
EPN species (S)	6	391.1	0.0001
L × T	10	3.3	0.0017
L × S	30	10.93	0.0001
T × S	12	29.1	0.0001
L × T × S	60	3.41	0.0001
Error	63		
Corrected total	188		

**Table 2:** Comparison of median lethal times (LT_50s_) for entomopathogenic nematode species (EPNs) against the various developmental stages of *Spodoptera frugiperda* larvae.

Larval Stages	EPNs	LT_50_ (h)	95% CI (Lower-Upper)	Slope (±SE)	χ^2^ (df)
First instar	*H. bacteriophora*	80 b	(50–141)	4.52 (1.13)	15.96 (1)
	*H. indica*	34 ab	(29–39)	5.26 (0.80)	43.64 (1)
	*S. arenarium*	84 bc	(72–99)	3.68 (0.66)	31.35 (1)
	*S. carpocapsae*	32 a	(28–36)	6.91 (1.12)	37.85 (1)
	*S. longicaudum*	57 ab	(19–103)	4.41 (1.19)	13.74 (1)
Second instar	*H. bacteriophora*	51 e	(44–57)	5.66 (0.79)	51.35 (1)
	*H. indica*	24 b	(na)	na	0.00 (1)
	*S. arenarium*	42 d	(36–46)	9.45 (1.83)	26.60 (1)
	*S. carpocapsae*	23 a	(na)	na	0.00 (1)
	*S. longicaudum*	26 c	(na)	na	0.00 (1)
Third instar	*H. bacteriophora*	64 ab	(23–115)	3.37 (0.90)	14.05 (1)
	*H. indica*	27 ab	(21–31)	5.07 (0.96)	28.14 (1)
	*S. arenarium*	27 ab	(24–31)	7.61 (1.59)	22.85 (1)
	*S. carpocapsae*	24 a	(na)	na	0.00 (1)
	*S. longicaudum*	28 ab	(23–31)	7.32 (1.58)	21.57 (1)
Fourth instar	*H. bacteriophora*	51 d	(44–57)	5.66 (0.79)	51.35 (1)
	*H. indica*	35 c	(31–40)	7.11 (1.09)	42.42 (1)
	*S. arenarium*	24 ab	(18–28)	5.77 (1.49)	14.95 (1)
	*S. carpocapsae*	28 b	(24–31)	6.34 (1.25)	26.01 (1)
	*S. longicaudum*	23 a	(na)	na	0.00 (1)
Fifth instar	*H. bacteriophora*	75 d	(64–89)	3.25 (0.58)	31.83 (1)
	*H. indica*	27 b	(24–31)	7.61 (1.59)	22.85 (1)
	*S. arenarium*	31 bc	(27–36)	6.58 (1.09)	36.54 (1)
	*S. carpocapsae*	25 ab	(20–30)	5.17 (1.04)	24.75 (1)
	*S. longicaudum*	23 a	(17–28)	5.19 (1.17)	19.70 (1)
Sixth instar	*H. bacteriophora*	75 d	(63–91)	3.04 (0.56)	29.77 (1)
	*H. indica*	46 c	(45–48)	36.79 (0.00)	0.00 (1)
	*S. arenarium*	31 ab	(27–36)	5.99 (0.96)	39.25 (1)
	*S. carpocapsae*	31 ab	(26–36)	6.00 (0.99)	36.97 (1)
	*S. longicaudum*	27 a	(20–33)	4.02 (0.70)	32.60 (1)

CI: confidence interval; na: not available. Corrected mortality was used to calculate LT_50_ values. LT_50_ values followed by the same letters are not significantly different (95% CI) among EPNs within the same larval stage.

**Table 3:** Reproduction rates (mean ± SE) of five entomopathogenic nematodes (EPNs) in the different larval stages of *Spodoptera frugiperda.* In a Petri dish, each EPN was applied at a density of 50 infective juveniles per larva in each *S. frugiperda* larval stage. Nematode numbers were then counted for 15 days after treatment.

Larval Stages	Nematode Reproduction (IJs/larva) (Mean ± SE)				
	Hb	Hi	Sa	Sc	Sl
L1	639 ± 123 Cb	2079 ± 282 Ca	858 ± 148C ab	1836 ± 456 Dab	981 ± 182 Dab
L2	2213 ± 225 Cb	4356 ±527 Ca	3308 ± 312 Cab	4523 ± 290 DEa	3431 ± 244 DEab
L3	4447 ± 401 Cb	7411 ± 819 Ca	5957 ± 480 Cab	7980 ± 854 Da	6597 ± 912 Dab
L4	14,083 ± 1433 BCab	30,963 ± 3961 Ba	24,987 ± 2031 Ba	26,200 ± 1010 Ca	25,706 ± 1244 Ca
L5	18,443 ±1499 Bab	47,930 ± 2323 Aa	24,988 ± 2031 ABa	36,537 ± 3002 Bbc	39,822 ± 1504 Bab
L6	30,308 ± 1789 Aab	58,753 ± 4047 Aa	35,867 ± 1975 Ab	58,433 ± 3349 Aa	57,990 ± 2837 Aa

EPNs: *Heterorhabditis bacteriophora* (Hb), *H. indica* (Hi), *Steinernema arenarium* (Sa), *S. carpocapsae* (Sc), and *S. longicaudum* (Sl). L1–L6 represent the larval stages of *S. frugiperda*. The same uppercase letter in the same column indicates that there was no significant difference in reproduction rate between the larval stages; the same lowercase letter in the same row indicates that there was no significant difference in reproduction rate between the EPN species (*p* > 0.05, Tukey’s test).
